# The Excellent Anti-Tumour Strategy (CTGVT, OV-gene) and the Excellent Anti-Tumor Gene (IL-24)

**Published:** 2012-06

**Authors:** Xin-Yuan Liu

**Affiliations:** 1 *State Key Laboratory of Cell Biology, Institute of Biochemistry and Cell Biology, Shanghai Institute for Biological Sciences, Chinese Academy of Sciences, Shanghai, 200031, China;*; 2 *Xin Yuan Institute of Medicine and Biotechnology, College of Life Sciences, Zhejiang Sci-Tech University, Hangzhou 310018, China*

## INTRODUCTION

Cancer gene therapy and cancer virotherapy have been widely studied for the anti-tumour effect in the last decades, but there has been no major breakthrough in either of them. Gene therapy has been laureated as the top 10 science news of 2009 because of its application for single gene hereditary diseases, but not for multiple gene abnormal cancers. Ad-p53 (1) and H101 (2) (OV of adenovirus) have been licensed for marketing in China for cancer therapy which have shown some benefit, but their therapeutic effects are limited, since they were used in a strategy employing gene therapy or virotherapy each separately. In 1999, we combined gene therapy and virotherapy together which was named as Cancer Targeting Gene-Virotherapy (CTGVT) constructed by inserting an anti-tumour gene into an oncolytic viral vector (OV), it is actually an OV-gene therapy (3, 4). The CTGVT (OV-gene) has been persistently studied for more than 10 years in our lab and about 70 peer-reviewed papers have been published (5-13) with a rather highly respected IF such as Hepatology, Cell Research, Cancer Research, Molecular Therapy, Clinical Cancer Research and so on.

**Figure 1:**
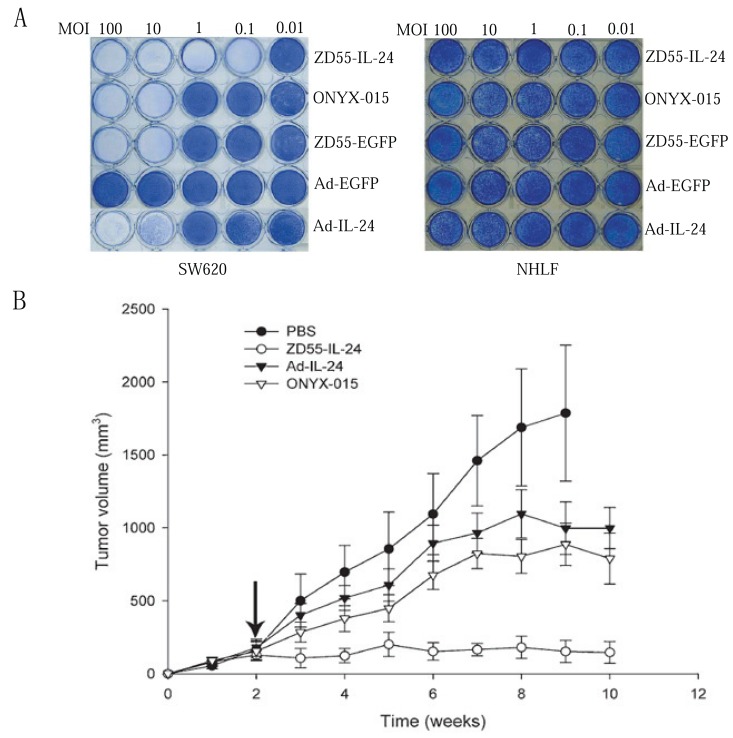
A, Tumor-selective cytopathic effect of ZD55-IL-24. Tumor cells SW620 and normal cells (NHLF) were seeded at a density of 1 × 10^5^ cells and infected with ZD55-IL-24, ONYX-015, Ad-EGFP, ZD55-EGFP, and Ad-IL-24 at the indicated MOIs. Seven days later, cells were stained with crystal violet; B, Antitumor activity of ZD55-IL-24 in SW620 xenograft model. Tumors were established by injecting SW620 cells subcutaneously into the right flank of nude mice. When tumors reached 100–150 mm^3^, the mice were divided into four groups (eight animals per group) and treated with four consecutive daily intratumoral injections of PBS or with ZD55-IL-24, Ad-IL-24, and ONYX-015 at 5 × 10^8^ PFU/dose per day (treatment indicated by arrow). Each time point represents the mean tumor volume for each group. Error bars represent the SEM. Tumor volumes were estimated as: tumor volume (mm^3^)=(width^2^×length)/2. Data are expressed as means of tumor volume over time (± SEM), n=8.

The CTGVT (OV-gene) is an excellent anti-tumour strategy. This is because that the oncolytic viral vector was used instead of replication deficient vector and the oncolytic virus can target on and replicate several hundred folds in cancer which leads to the inserted gene also being replicated several hundred-folds in cancer (14), therefore, the anti-tumour effect of CTGVT (OV-gene) is significantly increased. By inserting the interleukin-24 (IL-24) into the oncolytic adenovirus (OncoAd), the resulting OncoAd-IL-24, here is ZD55-IL-24 (ZD55 is an OV from adenovirus, i.e. the OncoAd) has much higher anti-tumour effect than that of Ad-IL-24 (Ad is a replication deficient adenovirus) *in vitro*, also higher anti-tumor effect than that of ZD55 (OV) and no toxicity to normal cells as shown in Fig. 1A (15). The anti-tumour effect by *in vivo* assay, the ZD55-IL-24 is also much higher than that of Ad-IL-24 (Fig. 1B). In summary of our circa 70 papers, the order of the anti-tumour effect is as below: OV-gene>OV≥Ad-gene (5-7). The recent crucial events of CTGVT (OV-gene) are: 1. The biotechnology giant Amgen paid 1 billion USD to purchase the OncoHSV-GM-CSF (OncoHSV is OV from Herpes simplex virus), which has potent anti-tumor effect (16). 2. A paper of OncoPox-GM-CSF (Poxvirus) has been published in Nature (17) because that OncoPox-GM-CSF is the first virus drug administed by intravenous injection and that OncoPox-GM-CSF can target to the metastasized tumor. All the above events validated that CTGVT (OV-gene) is an excellent anti-tumour strategy.

For achieving a strong anti-tumour effect, the use of potent anti-tumour genes are needed for the excellent anti-tumour CTGVT (OV-gene) strategy. The most potent (or one of the strongest) anti-tumour gene is IL-24 (interleukin-24) compared with more than twenty anti-tumour genes studied in our lab.

**Figure 2:**
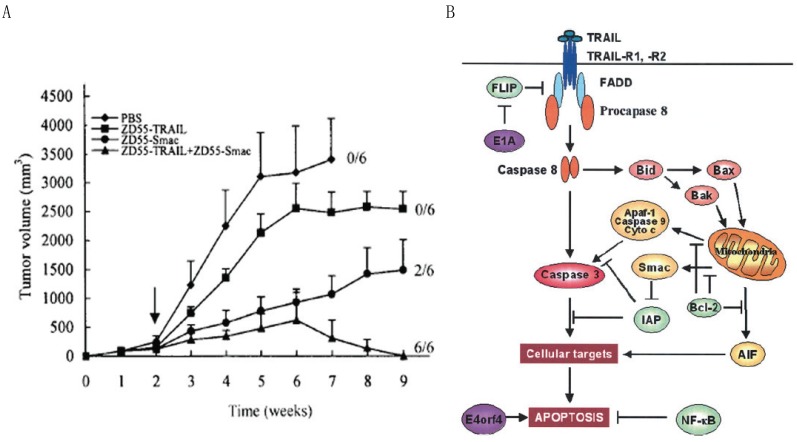
A Antitumor efficacy of ZD55-Smac and/or ZD55-TRAIL in nude mice bearing BEL7404 xenograft tumor. The HCC tumors were established by injection of BEL7404 cells subcutaneously into the right flank of nude mice. When the tumor reached 100–150 mm^3^, animals were treated with an intratumoral injection of 2 × 10^9^ pfu per animal of ZD55-TRAIL, ZD55-Smac, a combination of the two, or PBS as a control (arrow). The tumor size was measured and tumor volume was calculated. Data are expressed as means of tumor volume ± SEM (n=6). Abbreviations: PBS, phosphate-buffered saline; TRAIL, tumor necrosis factor–related apoptosis–inducing ligand; B Synergetic anti-tumor effect of ZD55-TRAIL and ZD55-IL-24. Apoptosis pathways can be initiated by death receptor ligation followed by receptor trimerization, recruitment of adaptor molecules (FADD), and activation of caspase-8. The activated caspase-8 activates caspase-3 and induces cell apoptosis. The activated caspase-8 also induces activation of Bid and regulates mitochondrial dysfunction. Cytochrome c alone with Apaf-1 activates caspase-9. Caspase-9 activates downstream caspases. These events finally result in apoptosis. Cancer cells may develop resistance to TRAIL through down-regulation of death receptors or caspase-8 and up-regulation of FLIP, Bcl-2, or IAP. Activation of NF-_B inhibits apoptosis. Overexpression of Smac results in degradation of IAP. Adenoviral proteins such as E1A inhibit FLIP accumulation by stimulating FLIP degradation in proteasome, or E4orf4 induces p53-independent cell death by apoptosis. TRAIL, tumor necrosis factor–related apoptosis–inducing ligand; Smac, second mitochondria-derived activator of caspases; IAP, inhibitor of apoptosis protein; FADD, Fas-Associated Death Domain; FLIP, FLICE (Fasassociated death-domain-like IL-1 beta converting enzyme)-inhibitory protein; AIF, apoptosis-inducing factor.

**Figure 3:**
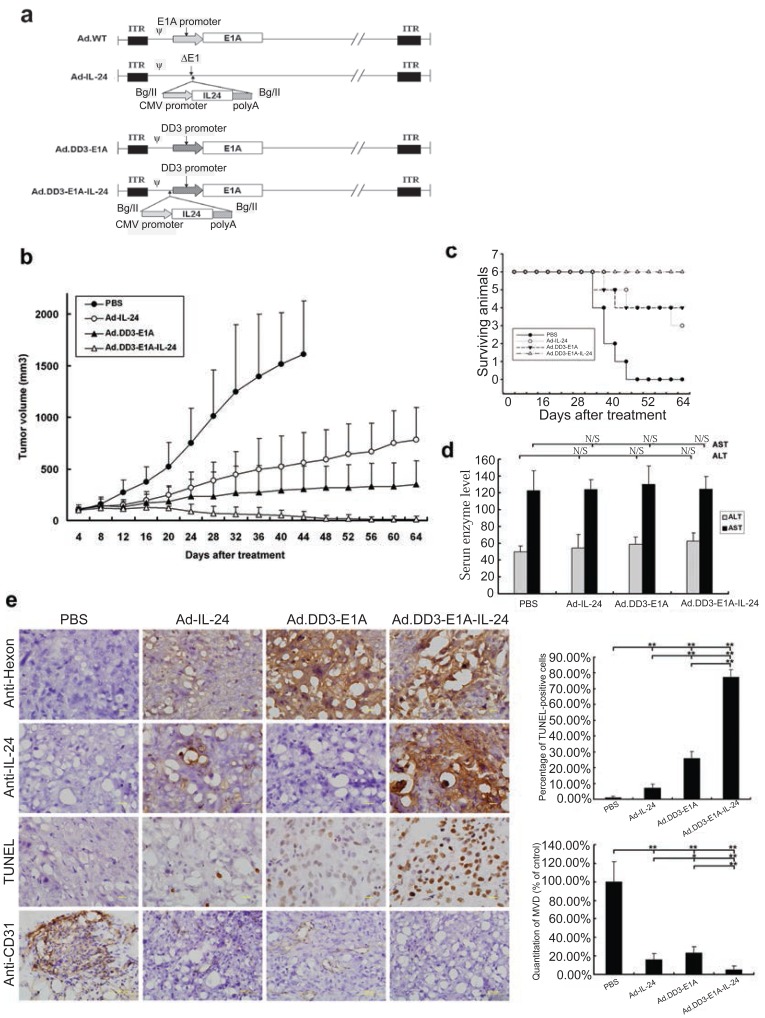
Antitumoral efficacy of the viruses in nude mice. PBS or different viruses were intratumorally administered to nude mice bearing DU145 xenograft tumors. (A) Construction of Ad•DD3•E1A•, (IL-24) is an expression cassettes; (B) The tumor volumes (mean 6 ± SD, n=6) were measured with caliper and estimated using the following formula: tumor volume (mm^3^) = length × width^2^/2; (C) The survival curve during the observation period; (D) The levels of serum enzymes ALT and AST are presented (NS means p>0.05, not significant); (e) Immunohistochemical staining with anti-hexon and anti-IL-24 antibodies to assess viral replication and the expression of the therapeutic gene, and TUNEL staining for the detection of apoptosis cells. Representative images from different samples at 400× magnification are shown. Scale bar=20 μm. Tumor sections were also stained with anti-CD31 by immunohistochemistry. Scale bar=100 μm. The proportion of TUNEL-positive cells as well as the MVD was quantified and presented as the mean 6 ± SD, respectively (n=5, **p<0.01; *p<0.05). [Color figure can be viewed in the online issue, which is available at www.interscience.wiley.com].

We have modified the CTGVT strategy by the combined use of two genes which was named as Cancer Targeting Dual Gene-Viro-Therapy (CTGVT-DG). Because that two gene may have compensative or synergetic effect, the CTGVT-DG strategy always could get complete eradication of all tumor xenograft (10-13, 18, 19). By the combined use of ZD55-TRAIL plus ZD55-Smac, all the hepatoma xenograft could be completely eradicated (Fig. 2A). This potent anti-tumor effect is due to synergetic effect between ZD55-IL-24 and ZD55-Smac (Fig. 2B) (10). Hepatoma usually content high IAP (Inhibitor of Apoptosis) which inhibit the function of caspase 3 and Smac can inhibit the function of IAP and activate caspase 3, that means the Smac can increase the apoptosis effect of TRAIL by block the function of IAP. However the TRAIL can induce the expression of caspase 8 which will induce Smac secretion through the mitochondrial pathway (Fig. 2B) (10). These showed the synergetic effect between ZD55-TRAIL and ZD55-Smac. Therefore, the combined use of ZD55-IL-24 and ZD55-TRAIL could get complete elimination of hepatoma xenograft. However, the CTGVT with only one gene still can’t very easily to eradicate all the xenograft tumor with the exception of IL-24 gene which has the excellent anti-tumor effect. For example, Ad•DD3•E1A•IL-24 could completely elimination of the xenograft prostate cancer as shown in Fig. 3A (here it is only one gene used in the CTGVT strategy). DD3 (differential display code3), a prostrate specific promoter was constructed as oncolytic viral vector (Ad•DD3•E1A) to drive IL-24 and construct Ad•DD3•E1A•(IL-24) (Fig. 3A), which has the excellent anti-tumor effect and could completely eradicate the xenograft praste cancer (Fig. 3B) (20). The so-called excellent anti-tumour effect used here by us means that the drugs could not only inhibit up to 100% of the xenografted tumour growth, but also eradicate the original growing and existing xenografted tumour completely without a treated nude mouse death as shown in Fig. 3B, 3C. The evidence for anti-tumor effect due to Ad•DD3•E1A•(IL-24) was shown in Fig. 3D. Similar complete elimination of xenograft hepatoma was obtained by the use of IL-24 only in the construction of Ad•AFP•E1A•ΔE1B•IL-24 (data not shown because that this paper is still under review). Therefore, IL-24 is an excellent anti-tumour gene which compare with two gene in the CTGVT-DG strategy and completely eradicate the xenograft tumor. The other example for the excellent anti-tumor effect of IL-24 is the combination of ZD55-IL-24 plus ZD55-TRAIL in which all the xenografted BEL 7404 hepatoma could be completely eradicated (Fig. 5A) since ZD55-IL-24 could up-regulate the expression of TRAIL (21) (Fig. 5C and ZD55-TRAIL could enhance the apoptotic effect of ZD55-IL-24 (21) as shown in Fig. 5B. All the above data showed that IL-24 is really an excellent anti-tumor gene.

**Figure 4:**
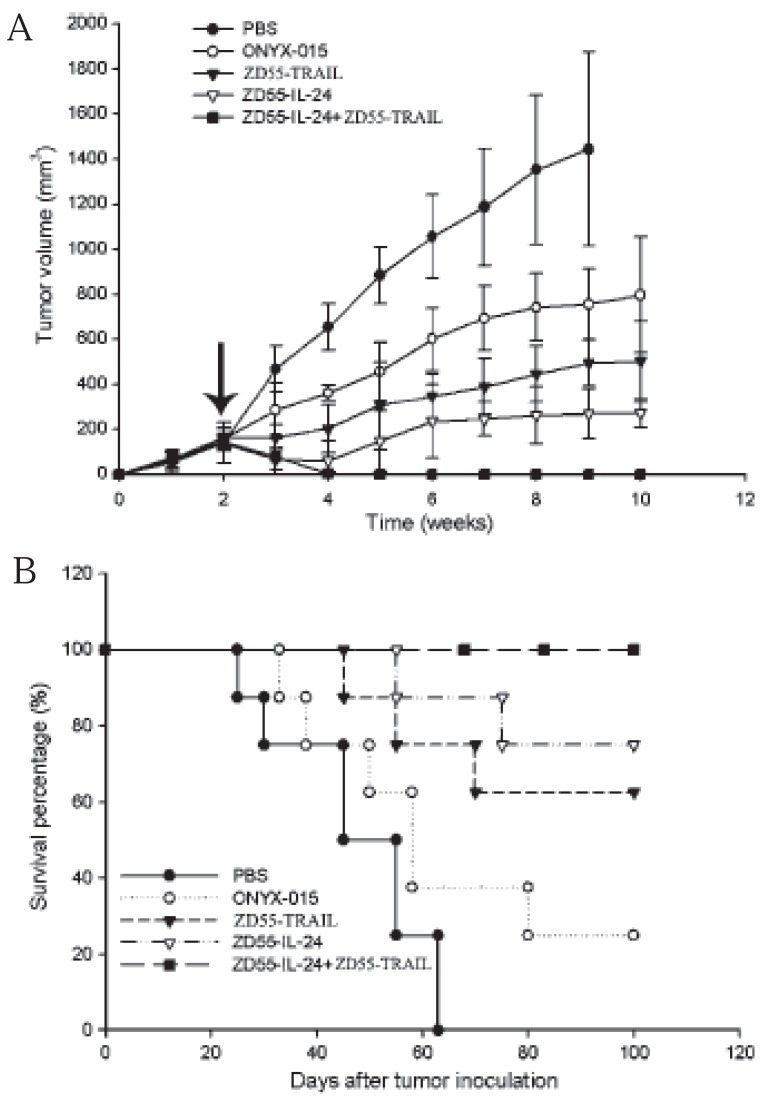
Complete eradication of human SW620 xenograft tumor in nude mice by the coadministration of ZD55-IL-24 and ZD55-TRAIL. When tumor size reached 100–150mm^3^, subcutaneous tumor-bearing mice were divided into four groups and treated with four consecutive daily intratumoral injections of PBS or with ZD55-IL-24, ZD55-TRAIL and the combination at 5 × 10^8^ PFU/dose per day (treatment indicated by arrow). (A) The tumor size was measured using calipers and tumor volume was calculated. Data are presented as means of tumor volume ±s.d. (n=8); (B) The death of animals was monitored. Long-term survival of animals was observed after treatment with oncolytic adenoviruses compared with control animals receiving saline.

**Figure 5:**
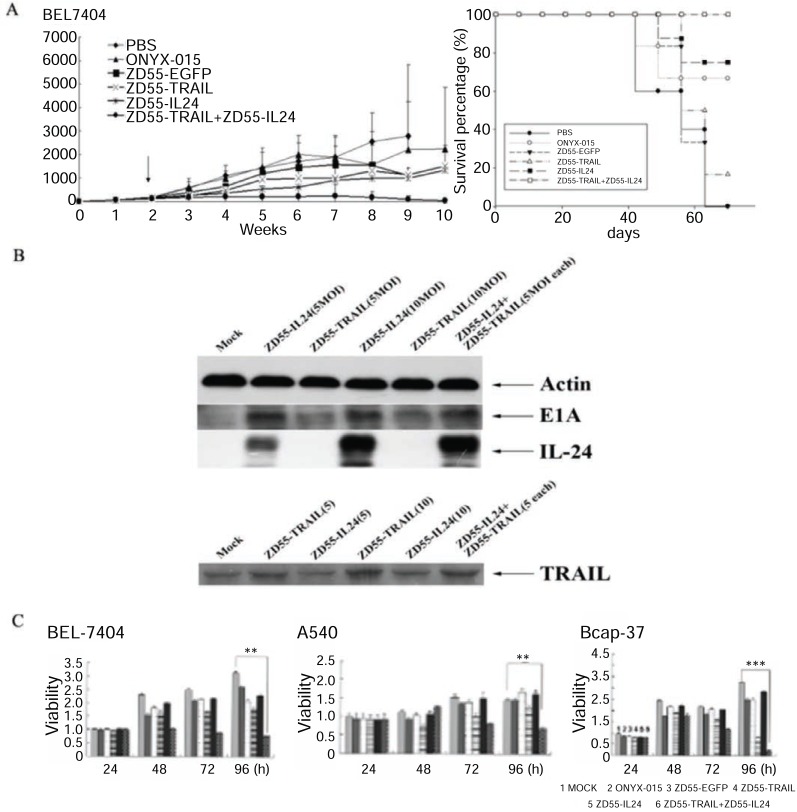
Synergatic effect of ZD55-TRAIL and ZD55-IL-24. (A) BEL-7404 was inoculated at right flank of nude mice. When the tumor volume grew to 100-120 mm^3^, mice were divided randomly into 5 groups, then different viruses were injected intratumorally every other day, 4 times with total amount of 2 × 10^9^ PFUs per mouse. The tumor volume were measured weekly after CTGVT viruses injection and also recorded the animal survival percentage. The arrow indicated the time of treatment. BEL-7404 for control groups (including PBS, ONYX-015 and ZD55-EGFP), n=6; (B) ZD55-IL-24 could up regulation the expression of TRAIL. (C) ZD55-TRAIL could enhance the apoptosis of ZD55-IL-24.

In short for getting excellent anti-tumor effect, the complete elimination of xenograft tumor, both the excellent vector and the excellent gene is all crucially needed. With the same IL-24 gene, Ad•DD3•E1A•(IL-24) or Ad•AFP•E1A•ΔE1B•(IL-24) could completely eliminate xenograft tumor, but the Ad•AFP•E1A•E1B(Δ55)•IL-24 could not (22), showing the importance of vector. With the same vector ZD55, the anti-tumor effect of ZD55-IL-24 is higher than that of ZD55-TRAIL (Fig. 4 in this paper, another evidences are not shown), showing the importance of gene. After combination of IL-24 and TRAIL, excellent anti-tumor gene TRAIL-L-IL-24 (L is linker) and excellent anti-tumor effect will be obtained to complete eradication of xenograft tumor (submitted).

We usually could get complete elimination of xenograft tumor, I am sure we can make drugs from modified CTGVT (OV-gene) such as CTGVT-DG, with (or for) an anti-tumour effect, higher than that of either 1 billion OncoPox-GM-CSF or Nature paper’s OncoPox-GM-CSF. These comparisons will be available soon later.
